# *In vitro* assessment of cytotoxic activities of *Lachesis muta muta* snake venom

**DOI:** 10.1371/journal.pntd.0006427

**Published:** 2018-04-16

**Authors:** Stephanie Stransky, Fernanda Costal-Oliveira, Letícia Lopes-de-Souza, Clara Guerra-Duarte, Carlos Chávez-Olórtegui, Vania Maria Martin Braga

**Affiliations:** 1 Departamento de Bioquímica e Imunologia, Instituto de Ciências Biológicas, Universidade Federal de Minas Gerais, Belo Horizonte, Minas Gerais, Brazil; 2 Centro de Pesquisa e Desenvolvimento, Fundação Ezequiel Dias, Belo Horizonte, Minas Gerais, Brazil; 3 National Heart and Lung Institute, Faculty of Medicine, Imperial College London, London, United Kingdom; Instituto de Biomedicina de Valencia, SPAIN

## Abstract

Envenomation by the bushmaster snake *Lachesis muta muta* is considered severe, characterized by local effects including necrosis, the main cause of permanent disability. However, cellular mechanisms related to cell death and tissue destruction, triggered by snake venoms, are poorly explored. The purpose of this study was to investigate the cytotoxic effect caused by *L*. *m*. *muta* venom in normal human keratinocytes and to identify the cellular processes involved in *in cellulo* envenomation. In order to investigate venom effect on different cell types, Alamar Blue assay was performed to quantify levels of cellular metabolism as a readout of cell viability. Apoptosis, necrosis and changes in mitochondrial membrane potential were evaluated by flow cytometry, while induction of autophagy was assessed by expression of GFP-LC3 and analyzed using fluorescence microscopy. The cytotoxic potential of the venom is shown by reduced cell viability in a concentration-dependent manner. It was also observed the sequential appearance of cells undergoing autophagy (by 6 hours), apoptosis and necrosis (12 and 24 hours). Morphologically, incubation with *L*. *m*. *muta* venom led to a significant cellular retraction and formation of cellular aggregates. These results indicate that *L*. *m*. *muta* venom is cytotoxic to normal human keratinocytes and other cell lines, and this toxicity involves the integration of distinct modes of cell death. Autophagy as a cell death mechanism, in addition to apoptosis and necrosis, can help to unravel cellular pathways and mechanisms triggered by the venom. Understanding the mechanisms that underlie cellular damage and tissue destruction will be useful in the development of alternative therapies against snakebites.

## Author summary

In this work, we investigate cellular events and mechanisms involved in *in cellulo* envenomation by *Lachesis muta muta* snake, which is one of the snakes responsible for the ophidic accidents in Brazil. Since the venom pathological effects are related to local symptoms such as edema and tissue necrosis, we evaluate venom action on normal human keratinocytes, skin cells directly affected during envenomation. Our data show the chronological analysis of cellular events triggered by *L*. *m*. *muta* venom, which stimulates autophagy, affects mitochondrial membrane potential and activates mechanisms that lead to cell death by apoptosis and necrosis. Crude venom also causes cell morphology and epithelial colony alterations. These findings give the first evidence about which cell death mechanisms are elicited by *L*. *m*. *muta* venom in skin cells. Understanding the pathways that underlie cellular damage will be useful to explain some of the pathological effects observed in local envenomation.

## Introduction

Snakebite is still a worldwide health problem and according to the World Health Organization (WHO), around 5.4 million people are bitten by snakes, resulting in 400,000 amputations and more than 125,000 deaths each year [1, 2]. Snakes from the genera *Bothrops*, *Lachesis*, *Crotalus* and *Micrurus* are responsible for the majority of envenomation cases in Brazil [3]. Snakes belonging to *Lachesis* genus (family Viperidae), known as bushmasters, are the largest venomous snakes inhabiting Central and South America and are divided into four species: *L*. *stenophys*, *L*. *melanocephala*, *L*. *acrochorda* and *L*. *muta*, which comprehends the subspecies *L*. *m*. *rhombeata* and *L*. *m*. *muta* [4, 5].

*Lachesis muta muta* is found preferentially in primary forests, including the Amazon, and despite being infrequent, human envenomation by this snake is considered severe due to its potential of injecting considerably large venom amounts (200–400 mg) [4, 6–9]. According to the Brazilian Ministry of Health, in the year 2015, this genus was responsible for 4% of the envenomation incidents, with mortality rates around 40% [10]. Compared to other Viperidae species, *Lachesis* venom has lesser toxicity and lethal activity, but due to the great quantity inoculated during accidents, the effects can be extremely severe [11]. The main systemic pathological effects triggered by envenomation include spontaneous hemorrhage, nausea, vomiting, diarrhea, coagulation disorders, hypotension, cardiovascular shock and renal malfunction [12]. Local effects are also observed and are characterized by edema, hemorrhage, ecchymosis and necrosis, the leading cause of permanent disability [13, 14]. Observed symptoms are probably a consequence of the direct action of venom toxins, such as snake venom serine proteinases (SVSP), snake venom metalloproteinases (SVMP), L-amino acid oxidase (LAAO), phospholipases A_2_ (PLA_2_) and hyaluronidase, that interfere with coagulation cascade, normal hemostatic system and tissue repair [6, 15].

Serum therapy by antivenoms is the only effective treatment used to neutralize circulating venom toxins and, if administered early, is powerful against several of the systemic effects. However, the progression of local effects can continue despite antivenom therapy and once triggered, most of the established damage cannot be reversed [13, 16]. Clinical symptoms are of greater importance due to complications related to tissue necrosis in *L*. *m*. *muta* envenomings. Thus, understanding the cellular mechanisms of how the extensive necrosis comes about at the bite site will help to identify ways to prevent tissue destruction.

Toxic effects caused by snake venoms in cell culture have been investigated by several research groups in the last years. Studies reported that *L*. *muta* venom is cytotoxic to VERO (derived from African green monkey kidney) and MDCK (derived from Madin-Darby canine kidney) cells [9, 17]. However, the cellular pathways triggered by the venom and its toxins are still poorly understood. Investigating the mechanisms of action of crude venom allows to understand the synergistic action of its toxins and to produce relevant information on the pathways triggered, being more closely related to what happens in real accidents.

In this article, we present a comprehensive study concerning *L*. *m*. *muta* cytotoxicity and the involvement of cell death mechanisms using normal human keratinocytes. These cells have an essential role in skin biology and this tissue is commonly used to evaluate the toxicity of various agents [18], in addition of being remarkably affected in snakebite victims. Therefore, the determination of events that underlie cellular damage and tissue destruction will help to identify pathways perturbed by crude *L*. *m*. *muta* venom and thus, explain some of the pathological effects observed in local envenomation.

## Methods

### Venom

Crude venom from *Lachesis muta muta*, comprising a pool of venom extractions from different individuals, was provided by Fundação Ezequiel Dias (FUNED), located in Belo Horizonte, Brazil. The venom was lyophilized and kept at -20°C until its use. To perform the tests the venom was reconstituted in ultra-pure water and protein concentration was measured by Lowry [19] or BCA method (Thermo Scientific), according to manufacturer’s instructions.

### Cell culture

The cell lines VERO (from normal epithelial monkey kidney), EA.hy926 (from human umbilical vein) and HeLa (from human cervical adenocarcinoma) were obtained from the American Type Culture Collection (ATCC—USA). The cell line MGSO-3, derived from human breast cancer tissue, were kindly provided by Dr Goes group from UFMG [20]. All cell lines were maintained in Dulbecco’s Modified Eagle’s (DMEM, Sigma Aldrich), high glucose, supplemented with 10% fetal bovine serum (FBS, Thermo Scientific—HyClone), 0.2% gentamicin (Gibco by Life Technologies) and kept in controlled atmosphere (5% CO_2_ incubator at 37°C).

Normal human keratinocytes from neonatal foreskin were obtained from a private collection isolated in 1995 and frozen down. These cells were cultured on a monolayer of 3T3 fibroblasts (also obtained from a private collection) treated with mitomycin C (4 μg/mL—Sigma Aldrich), for at least two hours, and maintained in FAD medium (DMEM:F12, BioWittaker, Lonza) supplemented with 10% FBS (Sera Laboratories International), 1.8 mM CaCl_2_, 5 mM glutamine, 100 units/mL penicillin, 100 μg/mL streptomycin, 5 μg/mL insulin, 10 ng/mL epidermal growth factor, 0.5 μg/mL hydrocortisone (all Sigma Aldrich) and 0.1 nM cholera toxin (Quadratech Diagnostics). Cells were kept in controlled atmosphere (5% CO_2_ incubator at 37°C).

### Cell viability assay

Cell viability was analyzed by Alamar Blue (Thermo Scientific) assay, according to Damico *et al*., with modifications [9]. Prior to the assay, 1x10^4^ cells/well (VERO, EA.hy926, MGSO-3 and HeLa) were plated on 96-well plates (Nunc) and incubated in a humidified 5% CO_2_ incubator at 37°C for 24 hours. Keratinocytes were plated on a monolayer of 3T3 fibroblasts, previously treated with mitomycin C (4 μg/mL—Sigma Aldrich), at a density of 2.2 to 3.2x10^4^ cells/well and incubated in a humidified 5% CO_2_ incubator at 37°C until the colonies reached 60–80% confluence. Then, cells were incubated with different amounts of *L*. *m*. *muta* venom diluted in DMEM (VERO with 0.3–10 μg/mL; EA.hy926 and HeLa with 1.25–40 μg/mL; MGSO-3 with 0.6–20 μg/mL) or with different amounts of *L*. *m*. *muta* venom diluted in FAD medium (Keratinocytes with 0.6–20 μg/mL), containing 1% FBS, for 24 hours. After incubation period, the culture supernatant was removed and Alamar Blue, diluted in DMEM (10% v/v), was added to each well. The plates were incubated for 3 hours and then the produced fluorescence was measured using a fluorescence plate reader (Synergy 2, Bio-tek) at 540 nm for excitation and 590 nm for emission. Cell viability was determined by comparing values of fluorescence with the mean fluorescence of the control (without venom and considered as 100% viability). The software GraphPad Prism 5 was used to calculate venom half maximal Effective Concentration (EC_50_—amount of venom able to reduce 50% of cell viability) from cell viability experiments.

### Cell death assays

To perform apoptosis and necrosis assays, keratinocytes were plated on 60x15 mm plates (Nunc), on a monolayer of 3T3 fibroblasts, previously treated with mitomycin C (4 μg/mL—Sigma Aldrich), at a density of 1 or 2x10^5^ and maintained in a humidified 5% CO_2_ incubator at 37°C until the colonies reached 80% confluence. To Sytox Green staining, keratinocytes were seeded on glass coverslips in 4 or 24-well plates (Nunc) at a density of 2 to 3x10^4^ and maintained in a humidified 5% CO_2_ incubator at 37°C, until the colonies reached 60% confluence.

Cells were treated with 2x EC_50_ (i.e., concentration able to kill 100% of cells) of *L*. *m*. *muta* venom for 6, 12 and 24 hours or with a solution of 1μM staurosporine or 0.1% Triton X-100 for 3 hours or 50 μM FCCP (Carbonyl cyanide 4-trifluoromethoxy phenylhydrazone—Sigma Aldrich) for 15 minutes, as positive controls for apoptosis, necrosis or mitochondrial membrane depolarization, respectively. Venom and positive controls were diluted in FAD medium containing 1% FBS. Cells incubated only with FAD medium were used as a negative control.

### Staining

Cells were detached using trypsin-EDTA solution (Sigma Aldrich), followed by centrifugation at 800 rpm for 5 minutes. Then, 1x10^5^ cells, from each time point (6, 12 and 24 hours), were stained using Annexin V-FITC (1:500 –Chemometec), to assess apoptosis, and Hoechst 3334 (10 μg/mL—Chemometec) for 15 minutes. Cells were centrifuged at 2000 rpm for 2 minutes and were then stained with Propidium Iodide (PI, 5 μg/mL—Chemometec), to assess necrosis. To evaluate mitochondrial membrane potential, 1x10^6^ cells from each time point (12 and 24 hours) were stained using JC-1 (2.5 μg/mL—Chemometec). Cells were centrifuged at 2000 rpm for 2 minutes and were then stained with DAPI for 15 minutes. The samples were analyzed by flow cytometry (NC-3000, Chemometec). All procedures were carried out according to the manufacturer’s instruction.

To further investigate necrosis, cells were stained with a solution of 1x TBS (Tris-buffered saline) and 10% FBS containing 167 nM Sytox Green (Thermo Scientific), for 30 minutes at room temperature in the dark. In the next step cells were stained with DAPI (Sigma Aldrich) for 15 minutes at the same conditions. After staining, coverslips were mounted in mowiol (Calbiochem).

### Autophagy assay

Keratinocytes were seeded on glass coverslips in 4 or 24-well plate (Nunc), on a monolayer of 3T3 fibroblasts, previously treated with mitomycin C (4 μg/mL—Sigma Aldrich), at a density of 2 to 3x10^4^ and maintained in a humidified 5% CO_2_ incubator at 37°C. After colonies reached the number of 20 to 30 cells, keratinocytes were transfected with GFP-LC3 (microtubule associated protein 1 light chain 3) construct using Fugene transfected reagent (Promega), as per manufacture’s instruction. After 24 hours incubation, cells were treated with the calculated 2x EC_50_ of *L*. *m*. *muta* venom for 1.5, 3 and 6 hours (diluted in FAD medium containing 1% FBS) or with EBSS (Earle’s Balanced Salt Solution—Sigma) for 30 minutes, as a positive control for autophagy. Cells incubated with FAD medium containing 1% FBS were used as a negative control. Transfection efficiency was qualitatively evaluated using EVOS XL microscope on fluorescence mode (magnification 20x) (Thermo Scientific). Normal keratinocytes are difficult to transfect and we estimate transfection efficiency at 30%. Cells were then fixed, stained with DAPI, as described above, and mounted in mowiol (Calbiochem).

### Microscopy

Phase contrast images following cell viability assay were obtained using Evos XL microscope (magnification 20x) (Thermo Scientific).

Images of Sytox Green stained cells and autophagic cells (LC3-puncta) were acquired with an Olympus Provis BX51 microscope (magnification 20x), a SPOT RT monochrome camera and SimplePCI software (Hamamatsu). Randomly selected images were analyzed using FIJI software.

### Quantification

For quantification of necrosis, the channel containing nuclei stained by DAPI or Sytox Green were subjected to image threshold followed by “fill holes” and “watershed” functions to identify all nuclei from the image. Then, “analyze particle” function was used and the size of the particles was adjusted to 15–1000 in order to count the nuclei. The number of necrotic cells (stained with Sytox Green) was divided by the number of nuclei stained by DAPI, to obtain the percentage of necrotic cells.

For quantification of LC3 puncta, the channel containing puncta was subjected to “find edges” function followed by image thresholding to identify all puncta from the image, excluding other structures in the cell. “Analyze particle” function was used and the size of the particles was manually adjusted to 0.8–2 in order to count the puncta. The number of LC3 puncta was calculated per image and then divided by the number of transfected cells, to obtain the average of puncta per cell.

### Statistical analysis

Results were expressed as means ± standard error of the mean (SEM). The statistical analysis was performed with GraphPad Prism 5 software. Student’s *t* test was used to compare venom-treated group and its control. A one-way analysis of variance (ANOVA), followed by Bonferroni’s test, was used to compare treated groups (4 time points). Two-way ANOVA, followed by Bonferroni’s test, was used to compare treated groups and their respective controls. A value of *p* < 0.05 indicated significance.

## Results

**Fig 1:**
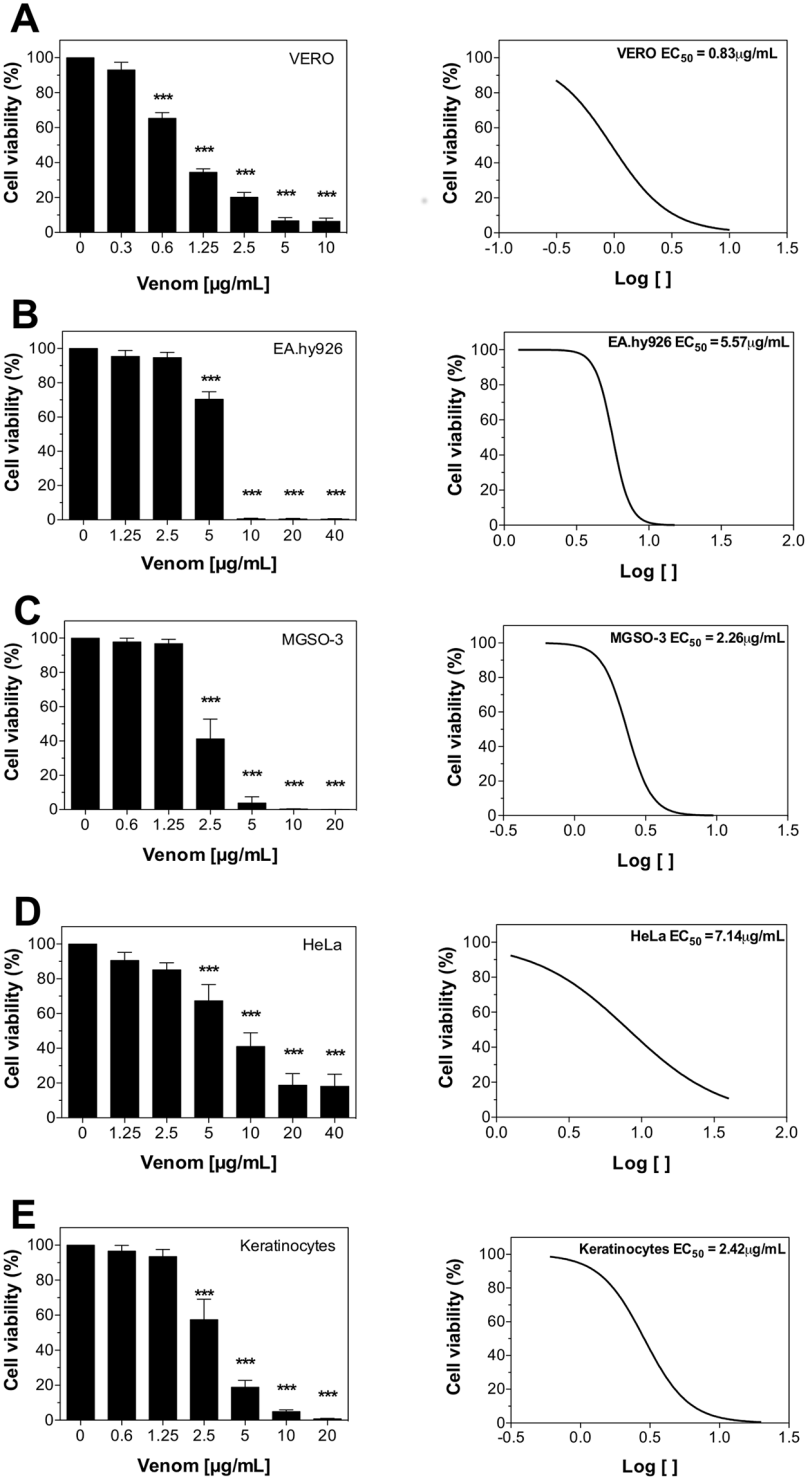
*Lachesis muta muta* venom cytotoxicity and EC_50_ curves against different cell lines. **(A)** VERO. **(B)** EA.hy926. **(C)** MGSO-3. **(D)** HeLa. **(E)** Keratinocytes. Cells were treated for 24 hours with serial dilutions of venom (0.3–40 μg/mL). Cell viability was analysed with Alamar Blue assay. The software GraphPad Prism 5 was used to calculate venom EC_50_. Data are represented as means ± SEM (n = 3, data were collected from three independent experiments). Venom-treated groups were compared using one-way ANOVA (***p < 0.001).

Venom cytotoxicity in different cells was evaluated after 24 hours using Alamar Blue reagent, which monitors the reducing environment of living cells and therefore evaluates metabolic function and cellular health [21]. This reagent is based on a fluorogenic redox indicator called resazurin, that is reduced to resorufin by viable cells metabolism in culture [22, 23]. *L*. *m*. *muta* venom decreased cell viability of tumor (MGSO-3 and HeLa), immortalized (VERO, EA.hy 926) and normal cells (keratinocytes) in a concentration-dependent manner. However, distinct levels of toxicity triggered by the venom were observed for each cell type (Fig 1). The relative cytotoxic activity was determined as the amount of venom capable of reducing 50% of cell viability or Effective Concentration (EC_50_). As shown in Fig 1, Vero cells were the most sensitive to the venom (EC_50_ = 0.83 μg/mL) followed by MGSO-3 (EC_50_ = 2.26 μg/mL) and keratinocytes (EC_50_ = 2.42 μg/mL), in comparison to EA.hy926 (EC_50_ = 5.57 μg/mL) and HeLa (EC_50_ = 7.14 μg/mL), which were more resistant. In addition, the venom response curve for VERO and HeLa cells did not follow a sigmoidal shape as those of other cell types. These results highlight the importance to identify the appropriate venom dosage when assessing cellular effects in different cell types.

**Fig 2:**
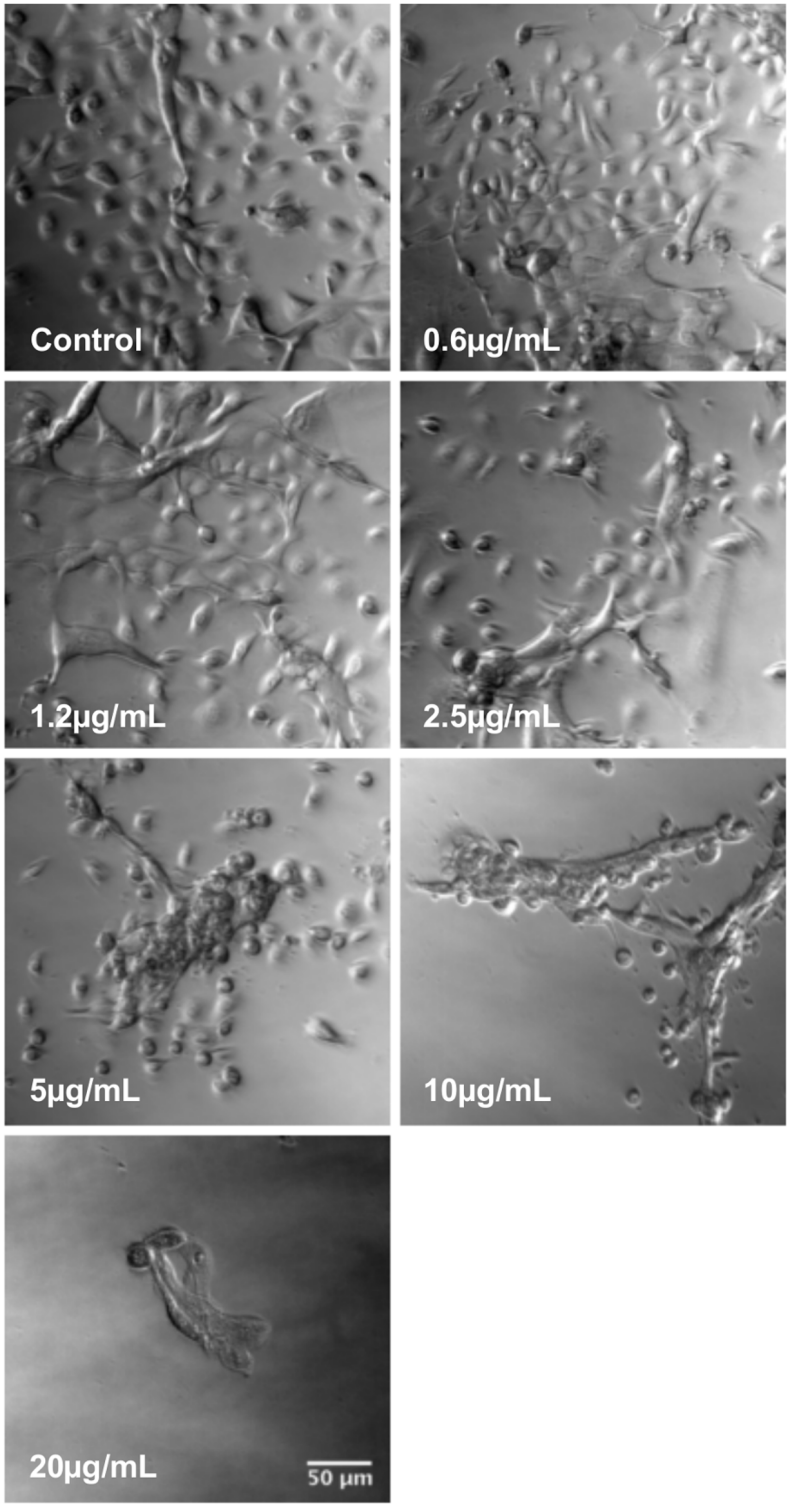
Morphological changes in keratinocytes after treatment with *L*. *m*. *muta* venom. Cells were treated for 24 hours with serial dilutions of venom (0.6–20 μg/mL). Control group was kept in culture medium for the same period of time. Images were acquired using widefield Zeiss Axio Observer microscope. Scale bar = 50μm.

Keratinocytes were selected for follow up studies, as they are normal human cells and thus could provide a response closer related to tissues affected by the venom at the bite site. These cells have been used previously to assess the cytotoxic effect and molecular mechanisms triggered by snake and spider venoms [24–26] and the therapeutic effect of bee venom [27]. Analysis of keratinocyte morphology treated for 24 hours with serial dilutions of *L*. *m*. *muta* venom (0.6–20 μg/mL) showed progressive cell retraction leading to colony collapsing after treatment with concentrations higher than 2.5 μg/mL (Fig 2).

**Fig 3:**
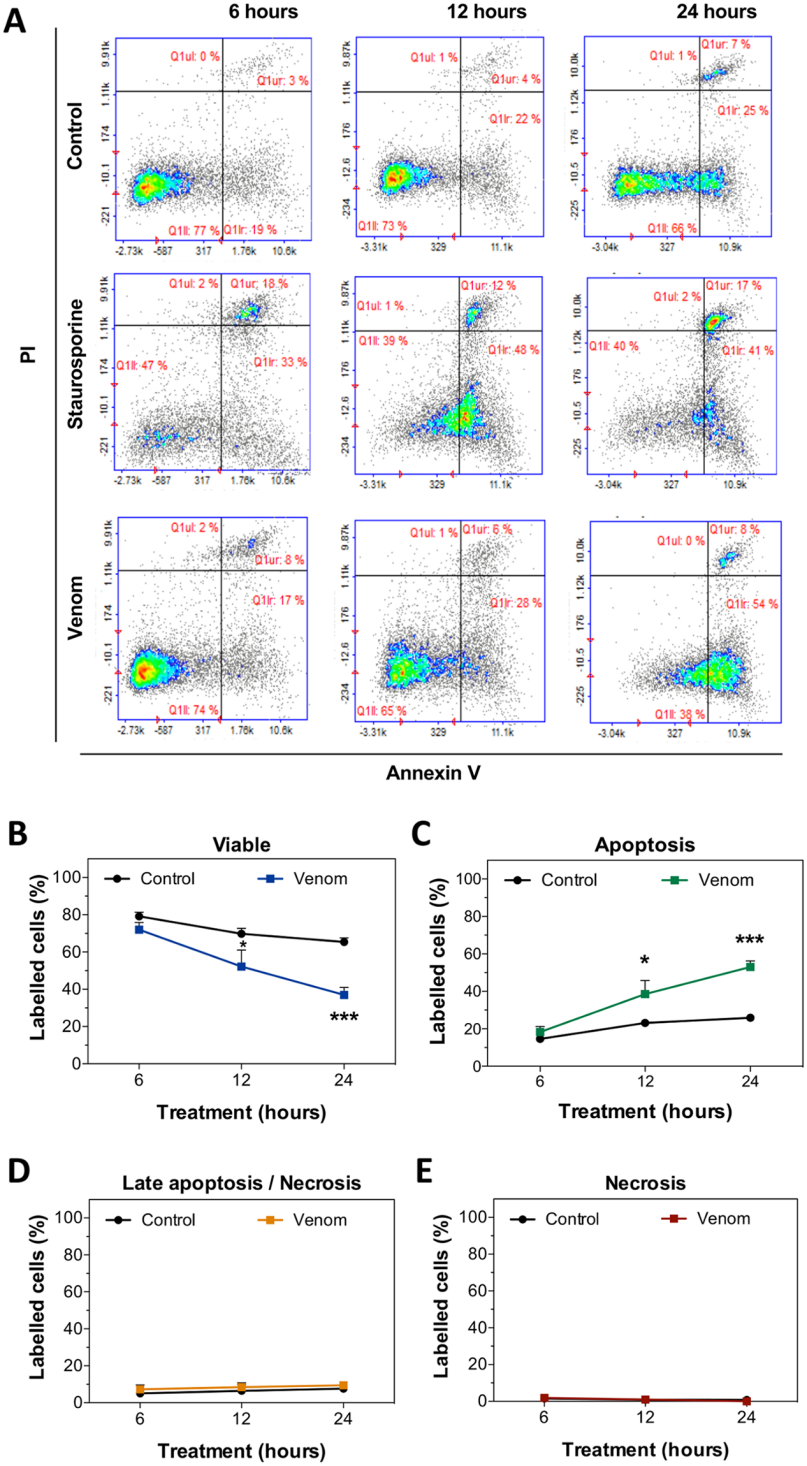
Apoptosis induced by *L*. *m*. *muta* venom in keratinocytes. **(A)** Representative cytometry plots for keratinocytes treated with staurosporine 1 μM for 3 hours, 2x EC_50_ of *L*. *m*. *muta* venom for 6, 12 and 24 hours, or left untreated. Cells were stained with Propidium Iodide (PI) and Annexin V-FITC. **B—E** Graphs show the percentages of viable cells **(B)**, apoptotic cells (labelled with Annexin V) **(C)**, cells undergoing late apoptosis or necrosis (double labelled with Annexin V and PI) **(D)** or necrotic cells (labelled with PI) **(E)**. Data are represented as means ± SEM (n = 3, data were collected from three independent experiments). Venom-treated groups were compared to their control using two-way ANOVA (*p < 0.05, ***p < 0.001).

To assess apoptotic and necrotic properties of the venom, we evaluated the levels of Annexin V-FITC and Propidium Iodide (PI) on human keratinocytes. Annexin V-FITC has been used to detect phosphatidylserine externalization during apoptosis, whereas PI detects necrosis by binding to DNA in necrotic cells when the cell membrane is compromised. Normal keratinocytes were treated for 6, 12 and 24 hours with 2x EC_50_ of *L*. *m*. *muta* venom (i.e., concentration able to kill 100% of cells) or for 3 hours with staurosporine, a well-known inducer of apoptosis. Cells were stained and analyzed by flow cytometry (Fig 3). Keratinocyte cultures, treated with venom for 12 and 24 hours, had reduced number of viable cells and a larger proportion of apoptotic cells (labelled with Annexin V) compared to untreated control cells (Fig 3A–3C). Using this assay, late apoptotic (double labelled) or necrotic cells (labelled with PI) were not observed after treatment with venom during the time frame tested (Fig 3D and 3E).

**Fig 4:**
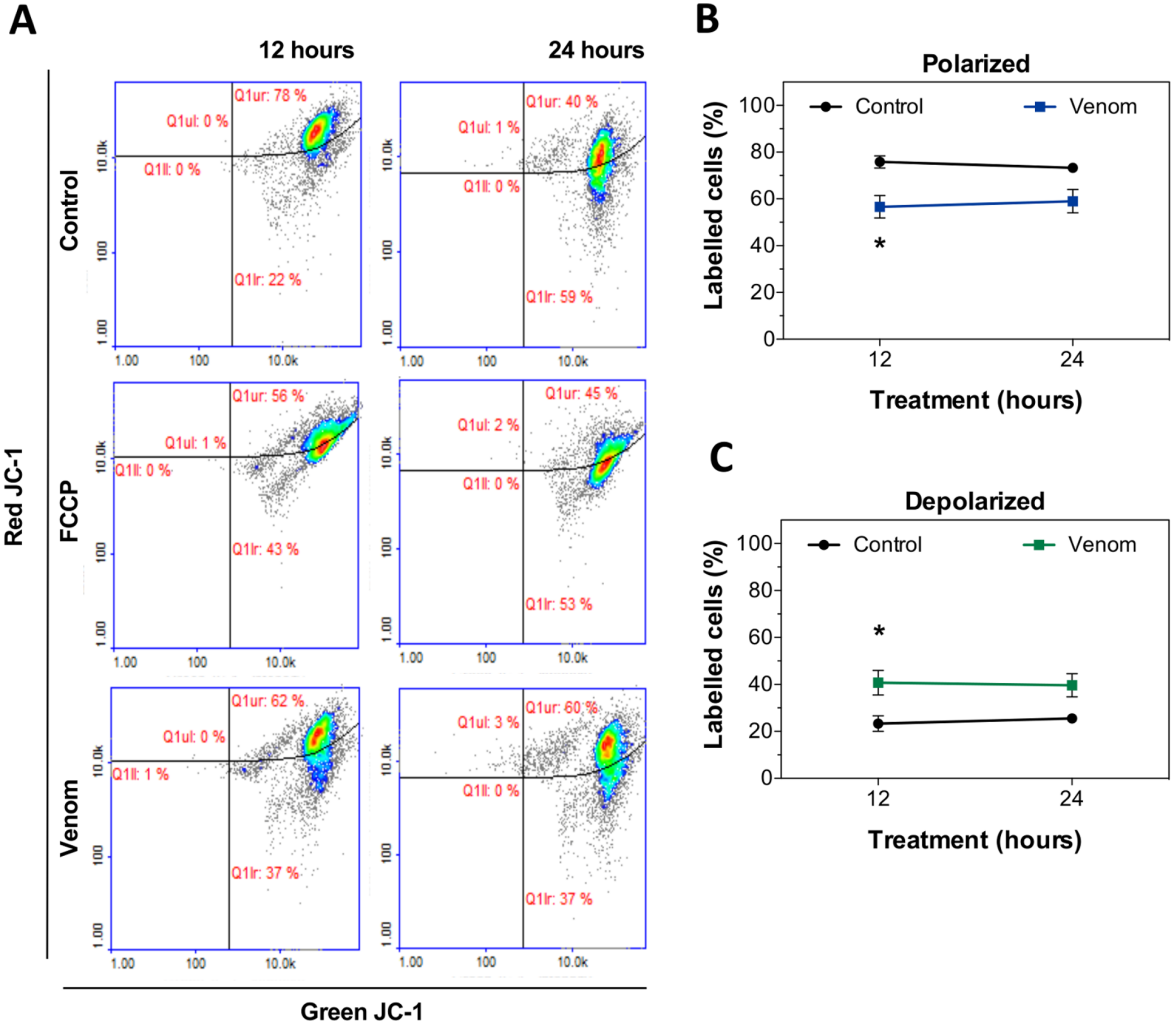
Mitochondrial membrane depolarization triggered by *L*. *m*. *muta* venom in keratinocytes. **(A)** Representative cytometry plots for keratinocytes mitochondrial membrane potential after treatment with FCCP 50 μM for 15 minutes, 2x EC_50_ of *L*. *m*. *muta* venom for 12 and 24 hours, or left untreated. Cells were stained with the dye JC-1. Graphs showing the percentages of normal (polarized) **(B)** and damage (depolarized) cells **(C)**. Data are represented as means ± SEM (n = 3, data were collected from three independent experiments). Venom-treated groups were compared to their control using two-way ANOVA (*p < 0.05).

Cellular events triggered during cell death by apoptosis include caspase activation, DNA fragmentation and mitochondrial membrane permeabilization. Mitochondrial alterations can be a decisive event between survival and cell death, as the permeabilization of the membrane can be responsible for the release of apoptogenic factors to initiate apoptosis [28]. To investigate the role of mitochondrial dysfunction in *L*. *m*. *muta* venom-induced cell death, keratinocytes were treated with venom for 12 and 24 hours or with FCCP for 15 minutes to assess depolarization of plasma and mitochondrial membrane. Cells were then stained with JC-1 dye. In normal cells, the negative charge established by the mitochondrial membrane potential allows accumulation of JC-1 in the mitochondrial matrix, as red fluorescent aggregates. In apoptotic cells, mitochondrial potential collapses and JC-1 locates in the cytosol in its monomeric green fluorescent form. After treatment for 12 hours with the venom, a significant decrease in the proportion of cells that have intact mitochondrial membrane (polarized) was observed (56%), compared to untreated control (75%) (Fig 4A and 4B). Conversely, a higher proportion of cells containing depolarized mitochondria was seen in venom-treated samples (Fig 4A and 4C). This effect was transient at 24 hours, as treatment of keratinocytes with venom did not trigger any changes in mitochondrial membrane potential (Fig 4A–4C).

**Fig 5:**
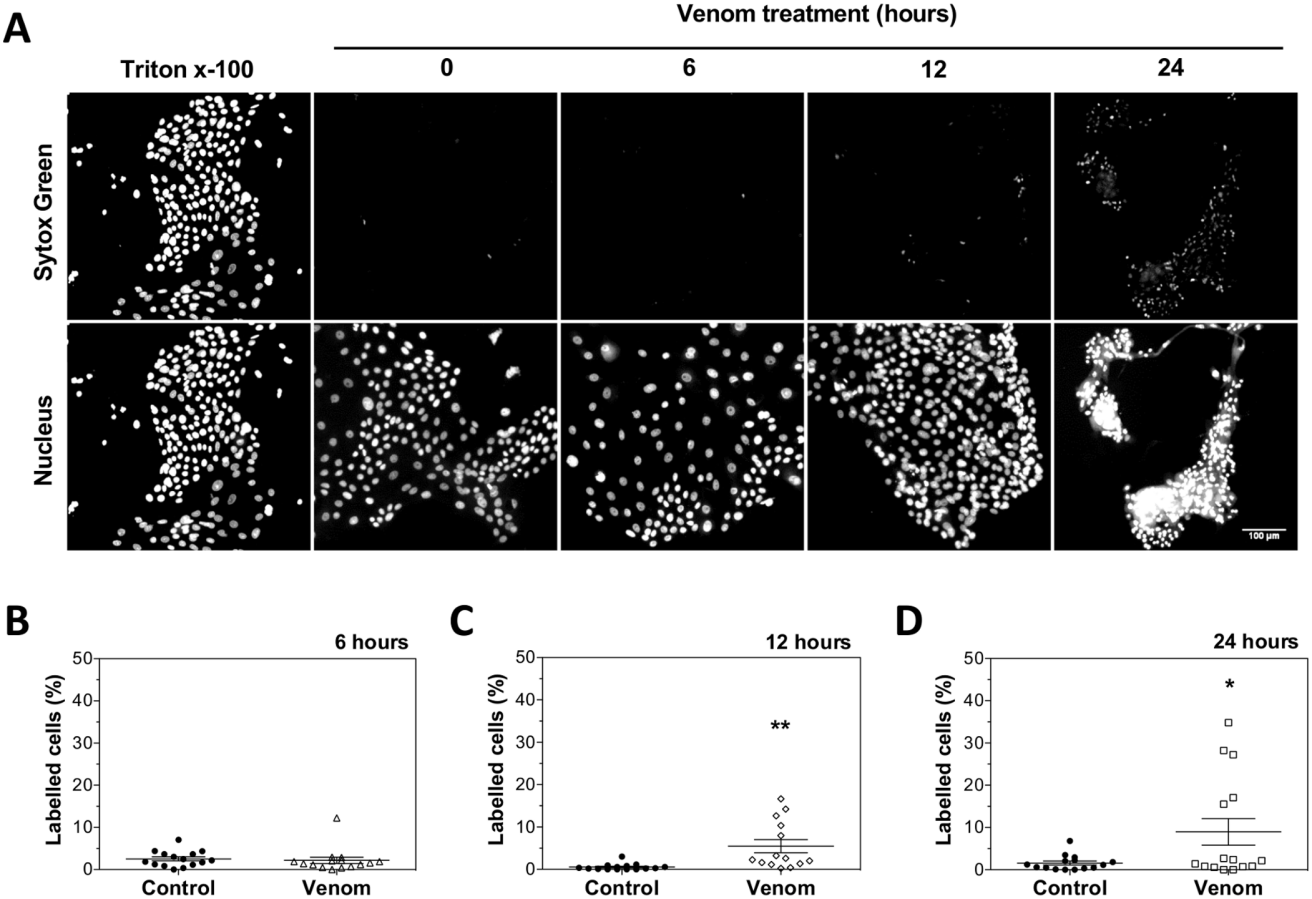
Necrosis induced by *L*. *m*. *muta* venom in keratinocytes. **(A)** Representative images of keratinocytes treated with 0.1% Triton X-100 for 30 minutes, 2x EC_50_ of *L*. *m*. *muta* venom for 6, 12 and 24 hours, or left untreated. Cells were stained with Sytox Green and DAPI to evaluate necrotic and normal cells, respectively. Scale bar = 100μm. Graphs showing the percentages of stained nucleus after treatment during 6 hours **(B)**, 12 hours **(C)** and 24 hours **(D)**. Data are represented as means ± SEM (n = 3, data were collected from three independent experiments). Venom-treated group was compared to control using Student’s t test (**p < 0.05, *p < 0.05).

To confirm our previous result that necrosis is not stimulated by venom treatment (Fig 3), a more sensitive probe, Sytox Green, was used. In necrotic cells, when the integrity of membrane is lost, the probe penetrates and binds to nucleic acids resulting in a >500-fold increase in fluorescence emission. Cells were treated with 2x EC_50_ for 6, 12 and 24 hours, stained with Sytox Green and analyzed by fluorescence microscopy. Treatment with venom for 6 hours did not induce necrotic events (Fig 5A and 5B). On the other hand, a significant increase in the proportion of cells stained by Sytox Green was observed after 12 and 24 hours of venom addition, when compared to untreated control (Fig 5A, 5C and 5D). By 24 hours, colony retraction and formation of cellular aggregates were also reproducibly observed (Fig 5A). Thus, using this more sensitive marker, we confirmed that necrosis occurs after 12 and 24 hours of *L*. *m*. *muta* venom treatment.

**Fig 6:**
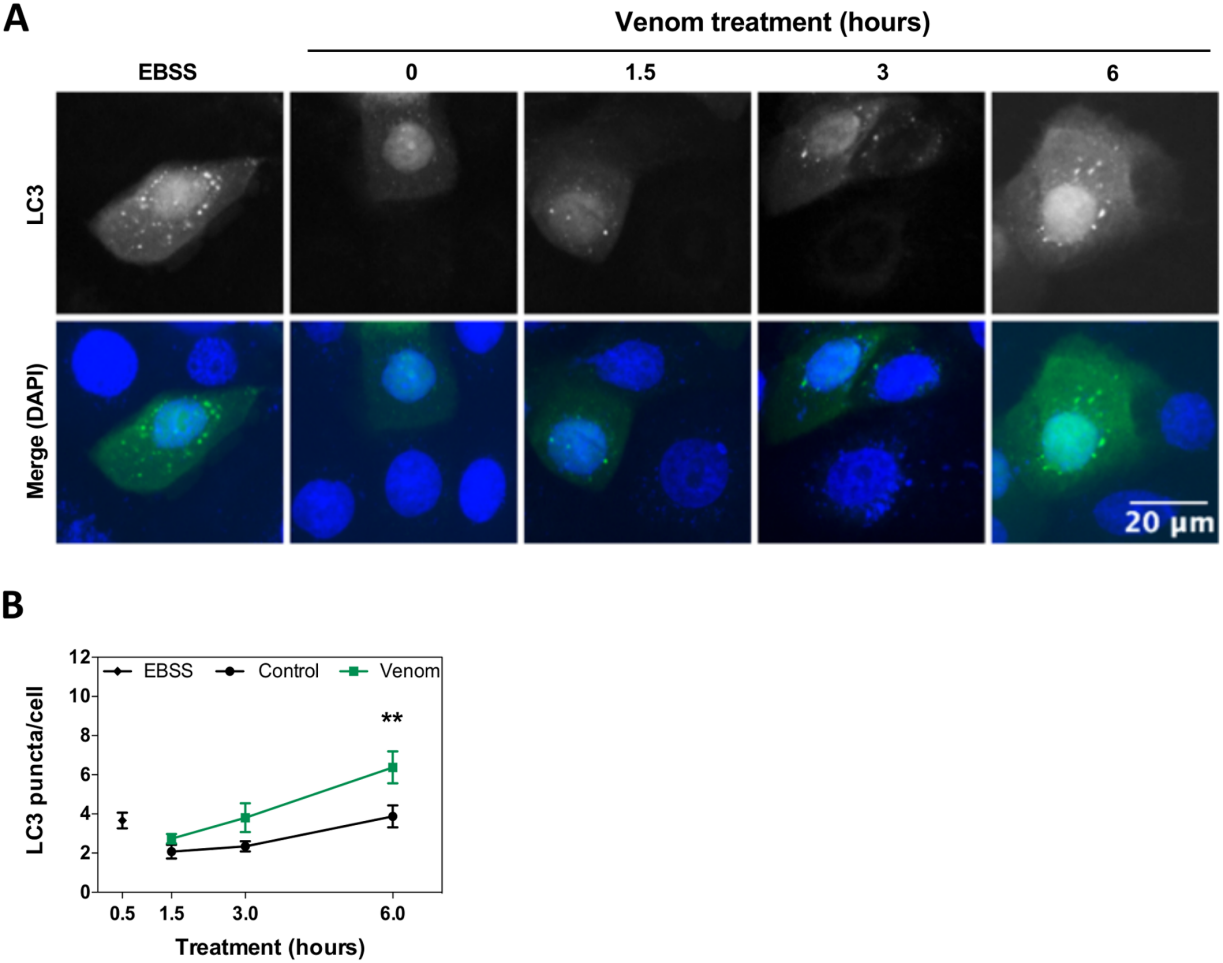
Autophagic events induced by *L*. *m*. *muta* venom in keratinocytes. **(A)** Representative images of autophagic vesicles and LC3 aggregation in keratinocytes starved using EBSS for 30 minutes, treated with 2x EC_50_ of *L*. *m*. *muta* venom for 1.5, 3 and 6 hours, or left untreated. Scale bar = 20 μm **(B)** Line graph showing the average of LC3 puncta per transfected cells after starvation or treatment for 1.5, 3 and 6 hours with venom. Data are represented as means ± SEM (n = 3). Venom-treated groups were compared using two-way ANOVA.

To evaluate if autophagy is activated in response to the cellular damage caused by crude venom, autophagosome formation was analyzed by transfection with GFP-LC3, a regulatory protein widely used to monitor autophagy. In normal, untreated cells, LC3 appears as a diffuse fluorescence signal in the cytoplasm. When cells are exposed to an autophagic stimulus, LC3 is cleaved into LC3 puncta and is recruited to the autophagosome membrane [29, 30]. Cells were treated with 2x EC_50_ for 1.5, 3 and 6 hours and analyzed by fluorescence microscopy. Data obtained revealed an increase in autophagosome formation (showed as number of LC3 puncta per cell) after treatment with venom for 6 hours, suggesting that autophagy plays a significant role in keratinocytes death by *L*. *m*. *muta* venom (Fig 6A and 6B).

Chronological analysis of cellular events during envenomation *in cellulo* showed that the venom stimulates autophagosome formation, depolarizes mitochondrial membrane and activates mechanisms that lead to cell death via apoptosis and/or necrosis. These events are accompanied by changes in cell morphology and retraction of the epithelial colony.

## Discussion

In developing countries, an important economical and societal impact of snake envenomation is the permanent damage due to scarring and potential amputation of affected limbs at the bite site [31]. This fact has received attention in the last years, with an increase in studies searching to unveil the cellular mechanisms involved in the pathogenesis of local envenomation [32–35]. In the present study, we report and describe the cytotoxic effect and cell death mechanisms triggered by *L*. *m*. *muta* venom on normal human keratinocytes, cells from epidermis, a tissue directly affected by lachetic accidents.

Cytotoxicity has been studied as a property of some viperid venoms using normal and cancer cell lineages [36–40]. Cytotoxic characterization studies have been previously performed by our group to demonstrate the toxic effects of *Bothrops* spp. venoms on VERO, MGSO-3 and HeLa cells [41, 42]. In the present study, exposure to *L*. *m*. *muta* venom also induced a significant decrease in viability of all lineages tested, but at different dosages (i.e. different EC_50_ values). While in our hands, cells were highly labile by exposure to *L*. *m*. *muta* venom in comparison with previous results [9, 17]. It is possible that the Alamar Blue test used herein is a more sensitive technique than those previously used. In addition, one also has to consider the distinct toxin stability and activities from different venom extraction and storage conditions.

An important aspect of envenomation is the pathological effects at the site of venom injection, which encompasses skin, connective tissue and muscle [4, 12, 43, 44]. More than 95% of epidermal tissue is composed of keratinocytes. These cells have been used for the study of skin related diseases, toxicity assessments [18] and an *in vitro* model for cutaneous loxoscelism (dermonecrosis) caused by spider venoms [25, 26]. Our data show that *L*. *m*. *muta* venom induces keratinocytes death via apoptosis and necrosis, which cannot be separated temporally. However, the proportion of apoptotic cells is higher than necrotic cells, suggesting that apoptosis may be the predominant form of cell death. It may be challenging to distinguish apoptosis from necrosis temporally, as the two processes can occur independently, sequentially or simultaneously. Furthermore, the mechanism of cell death (i.e. apoptosis or necrosis) caused by envenomation likely depends on the cell type and/or the extent (low or high doses) of stimuli [45, 46].

Biochemical events linked to apoptotic cell death include alterations of mitochondrial membrane permeabilization [47]. *L*. *m*. *muta* venom causes a transient mitochondrial membrane depolarization after treatment for 12 hours, suggesting the pore opening and release of pro-apoptotic molecules. Such events delimit the borderline between death or survival of cells and provide information about the intrinsic (mitochondrial) pathway of the apoptotic processes [48, 49]. On the other hand, extrinsic pathway involves activation of cell-death receptors on the cell surface via specific ligands [48]. Activation of extrinsic pathway leading to apoptosis has been reported following treatment with *Bothrops jararacussu* and *Bothrops asper* venom toxins [50, 51]. However, mass spectrometry of different snake venoms has not yet identified such ligands. It is possible that the extrinsic pathway could be activated indirectly in a paracrine manner rather than a specific action of a toxin in the venom. To address this issue, additional studies are necessary to investigate the potential role of toxins purified from *L*. *m*. *muta* venom in the activation of extrinsic apoptotic pathway.

Autophagy represents an essential function for cell homeostasis and adaptation in response to environmental alterations, such as starvation and clearance of intracellular proteins and abnormal organelles [29, 52]. The study of the ability of animal venoms to trigger autophagy is an emerging trend in toxinology field, and very few studies addressing this matter are found in the literature [53, 54]. Data presented here show an increase in the number of autophagic vesicles (autophagosomes) after treatment with *L*. *m*. *muta* venom. The timing of autophagy induction preceding venom-induced apoptosis/necrosis, indicate an unsuccessful, attempt by cells to cope with stress and restore homeostasis [47]. The dynamics in cell-death pathways triggered by a viperid venom is demonstrated here for the first time.

Crude venom is composed of several components, such as L-amino acid oxidases (LAAO), phospholipases A_2_ (PLA_2_), serine (SVSP) and metalloproteinases (SVMP) [6, 12]. The biochemical, immunological and *in vivo* properties of specific snake toxins have been extensively studied in the past. However, the cellular effects have been poorly explored so far. With respect to *L*. *m*. *muta* toxins, LAAOs are cytotoxic when tested in tumor cell lines (a gastric and breast cancer), while PLA_2_s do not reduce cell viability of MDCK (kidney) or C2C12 (skeletal muscle) [9, 55]. It is feasible that more than one toxin may contribute to disrupt cell homeostasis. Additional studies are necessary to identify which specific toxin(s) stimulate the autophagic, apoptotic and necrotic effects reported herein. However, as venom toxins act synergistically when injected to victims, as a result of targeting the same protein or biochemical pathway, understanding the effects caused by crude venom as a whole may be more adequate to elucidate the pathophysiology of local envenoming, enlightening the mechanism involved to suggest potential better treatments [56].

This study demonstrates that *L*. *m*. *muta* venom reduces cell viability in different cell lines and induces autophagy, apoptosis and necrosis in normal human keratinocytes. We speculate that autophagic events are probably triggered in an attempt to help to eliminate the toxic stimulus and to repair damaged cells. To the best of our knowledge these findings give the first evidence about which cell death pathways are elicited by *L*. *m*. *muta* crude venom in skin cells. Future studies will dissect which specific toxins from *L*. *m*. *muta* venom are responsible for triggering the temporally and mechanistically distinct modes of cell death shown herein: autophagy, apoptosis and necrosis.
